# Associations of 24-h movement behaviors with depressive symptoms among cancer survivors: findings from the NHANES 2011–2014

**DOI:** 10.1007/s11764-026-02034-0

**Published:** 2026-05-16

**Authors:** Vagner Deuel de O. Tavares, Megan Teychenne, Tiago V. Barreira, Felipe Barreto Schuch, Gary Skinner, Nicole Leite Galvão-Coelho, Eduardo Caldas Costa, Rodrigo Alberto Vieira Browne

**Affiliations:** 1 Faculty of Nursing, University of Calgary, 3395 Hospital Drive NW, Calgary, AB T2N 5G2 Canada; 2 Laboratory of Hormone Measurement, Department of Physiology and Behavior, Federal University of Rio Grande Do Norte, Natal, RN Brazil; 3 Institute for Physical Activity and Nutrition (IPAN), School of Exercise and Nutrition Sciences, Deakin University, Geelong, VIC Australia; 4 Falk College of Sport, Syracuse University, Syracuse, NY USA; 5 Department of Sports Methods and Techniques, Federal University of Santa Maria, Santa Maria, RS Brazil; 6 Institute of Psychiatry, Federal University of Rio de Janeiro, Rio de Janeiro, RJ Brazil; 7 Faculty of Health Sciences, Universidad Autónoma de Chile, Providencia, Chile; 8 NICM Health Research Institute, Western Sydney University, Westmead, New South Wales Australia; 9 National Institute of Science and Technology for Translational Medicine – INCT TM – CNPq/FAPESP/CAPES, Ribeirão Preto, Brazil; 10 Department of Physical Education, Federal University of Rio Grande Do Norte, Natal, RN Brazil; 11 Graduate Program in Physical Education, Catholic University of Brasília, Brasília, DF Brazil QS 07, Lote 01, EPCT, Taguatinga Sul - Taguatinga, 71966-700

**Keywords:** Moderate-to-vigorous physical activity, Physical behaviors, Sedentary behavior, Sleep, 24-h movement behavior, Depressive symptoms

## Abstract

**Background:**

Cancer survivors have a high prevalence of depressive symptoms. Moderate-to-vigorous physical activity (MVPA), sedentary time, and sleep are key modifiable behaviors associated with depressive symptoms. This study examines the associations between accelerometer-measured 24-h movement behaviors and self-reported depressive symptoms in cancer survivors.

**Methods:**

This is a cross-sectional analysis using data from the NHANES 2011–2012 and 2013–2014 cycles. The 24-h movement behaviors were measured by wrist accelerometers, including MVPA, sedentary time, and nightly sleep time. Depressive symptoms were assessed using the Patient Health Questionnaire (PHQ-9). Robust Poisson regression was used to estimate the prevalence ratio (PR) with their respective 95% confidence intervals (CI) for the categorical depression outcomes, considering independent and combined analyses of the exposures.

**Results:**

Data from 683 adults (aged 65.0 ± 13.1 years, females 55.5%) were analyzed. In mutually adjusted analyses, compared with inactive cancer survivors (< 150 min/week), those who were active (≥ 150 min/week of MVPA) had lower depressive symptoms (PR = 0.70; p = 0.026). Sedentary and sleep time were not independently associated with depressive symptoms. Meeting the combined recommendations for both MVPA and sedentary time (PR = 0.77; p = 0.044) and for MVPA and sleep time (PR = 0.76; p = 0.041) was associated with fewer depressive symptoms. Similarly, meeting the combined recommendations for sedentary and sleep time (PR = 0.64; p = 0.009), as well as all three behavior recommendations simultaneously (PR = 0.64; p = 0.009), were associated with lower symptoms.

**Conclusion:**

MVPA was consistently associated with fewer depressive symptoms among cancer survivors, whereas sedentary and sleep time alone showed no independent association. Combined movement behaviors patterns, particularly were also associated with a lower prevalence of depressive symptoms, reinforcing the potential relevance of an integrated 24-hour movement behavior approach for mental health in this population.

**Implications for Cancer Survivors:**

Strategies that promote physically active lifestyles and other healthy (non)movement behaviors, including reduced sedentary time and adequate sleep time, may help support mental health among cancer survivors.

**Supplementary Information:**

The online version contains supplementary material available at https://doi.org/10.1007/s11764-026-02034-0.

## Background

The diagnosis of cancer is a challenging event that simultaneously impacts individuals psychologically, emotionally, and financially [1]. The impact of cancer on mental health is likely due to various factors, including side effects of cancer treatment medications (e.g. nausea, exhaustion), prolonged use of the healthcare system (adversely impacting life routine and finances), and impact on daily functioning (i.e., fatigue and pain), being in a state of prolonged physical ill-health, and loss of quality of life [2]. Depressive symptoms are particularly notable among cancer survivors, as they have been increasing worldwide among this population by around 33% [3].

Risk factors for depression generally include genetic, epigenetic, and biological (e.g., inflammation) factors, and environmental stressors (e.g., negative life events) [4]. A robust body of evidence highlights the protective role of physical activity, particularly at moderate-to-vigorous physical activity (MVPA) intensity, against depressive symptoms [5–7]. Furthermore, reductions in sedentary time (i.e., sitting or reclining behaviors during waking hours) have been associated with reduced depressive symptoms, whether reallocating time for physical activity or not [8–10], while sleep health (duration and quality) has been associated with a lower risk of depression [11]. However, evidence of the associations between these 24-h movement behaviors and depressive symptoms in cancer survivors is limited. For example, studies showed that physical activity was inversely associated with depressive symptoms in cancer survivors [6, 12]. Further, a small body of research shows a positive association between sedentary time and psychological symptoms in cancer survivors [6, 13]. In addition, prolonged sedentary time has been linked to various physical health problems (e.g., cardiovascular disease, inflammation, and insulin resistance) [14], which may in turn exacerbate depressive symptoms in cancer survivors [15]. Evidence also highlights an association between sleep disorders and greater severity of depressive symptoms in cancer survivors [16].

A previous scoping review including cross-sectional studies showed that among patients diagnosed with cancer, 24-h movement behaviors with time reallocation, that is, substituting one behavior for another, could improve health outcomes such as better body composition, faster cognitive processing, reduced fatigue, and better bone health [17]. Likewise, these 24-h movement behaviors may be associated independently or in combination with mental health outcomes of cancer survivors. Yet currently, there is a dearth of research examining the independent and combined associations of 24-h movement behaviors with depressive symptoms in cancer survivors.

Cancer survivors represent a challenging population that is generally less physically active [18], spends more time sedentary [19], and has poorer sleep health [18] compared with the general population. In the general population, 24-h movement behaviors related to depressive symptoms may modify their associations with depressive symptoms [20–22]. Findings derived from the general population, for which there is a substantial body of evidence, cannot be assumed to translate directly to cancer survivors, as survivorship often leads to changes in functional capacity, fatigue, pain, and psychosocial stressors that influence both movement behaviors and depressive symptoms.

To our knowledge, no previous study has examined the associations between 24-h movement behaviors and depressive symptoms in cancer survivors. Moreover, accelerometry assessments provide a more accurate and comprehensive characterization of movement behaviors across the 24-h cycle than self-reported measures and are therefore critical for advancing research in this field. Thus, this study aimed to examine the mutually adjusted and combined associations of adherence to accelerometer-measured 24-h movement behaviors with depressive symptoms in cancer survivors, consistent with the Canadian 24-Hour Movement Guidelines (i.e., ≥ 150 min/week of MVPA, < 8 h per day of sedentary behavior, and 7–9 h of nightly sleep). Since physical activity, sedentary time, and sleep time represent interdependent dimensions of human (non)movement across the 24-h cycle, this investigation may provide a greater understanding of the combined impact of these behaviors on depressive symptoms among cancer survivors.

## Methods

### Design study

This study employed a cross-sectional design using data from two consecutive cycles of the National Health and Nutrition Examination Survey (NHANES 2011–12 and 2013–14). NHANES uses a complex, multistage probability sampling strategy to obtain a nationally representative sample of the noninstitutionalized U.S. population. Data integration followed NHANES analytic guidelines, combining interview and examination data with accelerometer-measured 24-h movement behaviors and depressive symptoms among adults with a history of cancer. Self-reported data were collected during the Mobile Examination Center (MEC) visit, followed by an accelerometry assessment.

### Participants

The NHANES sample represents the total noninstitutionalized civilian U.S. population residing in the 50 states and the District of Columbia. NHANES aims to collect comprehensive individual-level health data in biennial cycles through structured questionnaires, physical examinations, and laboratory protocols. All participants provided written informed consent, and the CDC and the National Center for Health Statistics Ethics Review Board approved the study protocols. The 2011–12 and 2013–14 cycles are the most recent NHANES cycles with objective measurement of movement behaviors among cancer survivors.

### 24-h movement behaviors

Accelerometer-measured movement behaviors were assessed within a 24-h movement framework based on the Canadian guideline [23]. The Canadian guideline included sedentary time, nightly sleep time, and MVPA. Sedentary time was classified into two categories: more than 8 h/day and fewer than 8 h/day. Nightly sleep time was categorized as inadequate (< 7 or > 9 h per night) or adequate (7–9 h per night). MVPA was grouped into two categories based on weekly minutes of activity: < 150 min/week (inactive) and ≥ 150 min/week (active). Data were obtained from each participant through continuous use of an accelerometer for nine consecutive days. Participants began wearing the monitor immediately after their initial examination at the Mobile Examination Center (MEC) and removed it on the morning of the ninth day. The device was worn on the nondominant wrist using an adjustable mesh strap, and participants were instructed to wear it continuously throughout the monitoring period. The ActiGraph GT3X + was selected for its triaxial measurement capabilities, long battery life, and water resistance, see more details [24]. Raw data were downloaded, and time spent in the different movement behavior classifications was computed from the raw accelerometer signals to ENMO (mg) values using the GGIR R package version 3.2.8 [25–28]. No changes were made to the package settings to facilitate easier replication of the accelerometer data processing. Data were summarized based on 5-s epochs and non-wear time was calculated based on the standard deviation (< 3 mg) and value range (< 50 mg in two of three-axis) of the accelerometer axis, using the acceleration windows of 60 min with 15 min increments [26, 27]. Activity intensities were classified using the following cut-points for sedentary (< 40 mg), light (40–100 mg), moderate (100–400 mg), and vigorous (> 400 mg). A measurement day was defined from midnight to midnight. Days with fewer than 16 h of device wear time were removed, as were observations originally flagged as invalid and all values above the 99th percentile. A minimum of four valid days was required [29]. Average values from valid days were used in the analysis.

### Depressive symptoms

For depressive symptoms, the Patient Health Questionnaire-9 (PHQ-9) from the NHANES survey was used. This is a self-reported, open-access, and validated instrument consisting of nine items based on the DSM-IV diagnostic criteria for major depression. Each item ranges from 0 (never) to 3 (almost every day), with total scores ranging from 0 to 27. The PHQ-9 was used both as a continuous measure [30] and as a categorical variable divided into two groups: no depressive symptoms (0–4) and having depressive symptoms (≥ 5) [31].

### History of cancer

The NHANES questions related to cancer history were self-reported. Participants responded to standardized items, including: “Ever told you had cancer or malignancy?” and, for those who answered affirmatively, “What kind of cancer?”. After participants reported the type(s) of cancer, additional questions were collected regarding age at first diagnosis for each reported cancer type. In NHANES, age at diagnosis is reported separately for each cancer type. The primary cancer was defined as the cancer with the earliest reported age at diagnosis. Time since cancer diagnosis was calculated as the difference (in years) between age at interview and age at diagnosis.

### Sociodemographic information

Sociodemographic variables included age, sex (male/female), alcohol use in the past 12 months (yes/no) and lifetime use, body mass index (BMI) (normal weight, BMI ranging from 18.5 to 24.9 kg/m^2^; overweight, BMI ranged from 25.0 to 29.9 kg/m^2^; and obesity, BMI ≥ 30 kg/m^2^), education level (low education, high school, or higher education), income (low income (0–34 k), middle income (35–74 k), and high income (> 75 k) [32], citizenship (yes/no), and marital status (married/common-law, widowed, divorced/separated, or never married).

### Statistical analysis

Continuous variables were described as mean ± standard deviation (SD), while categorical variables were described as absolute (n) and relative (%) frequencies. Poisson regression with robust variance was used determine prevalence ratio (PR) and 95% confidence interval (CI) for depressive symptoms (cutoff < 4 for no depression and ≥ 5 for depression), considering MVPA, sedentary time, and nightly sleep time independent for unadjusted model and mutually adjusted model, including the following variables: age, sex, BMI, income, education and alcohol use. Additionally, combined analyses were performed for each pair of movement behaviors and for all three behaviors simultaneously. For the pairwise comparisons, participants who met both recommendations were compared to those who met one or none of the respective behaviors, with additional adjustment for the remaining movement behavior. For the three-behavior model, participants meeting all recommendations were compared to those meeting two or fewer behaviors, adjusting for the same covariates. In addition, sensitivity analyses excluding non-melanoma skin cancer survivors were conducted to assess potential bias. Model assumptions, including multicollinearity, were assessed. The quality of fit of the models was assessed using the Omnibus test. A p-value < 0.05 was considered statistically significant for all analyses. All analyses were conducted using SPSS v.27 software (IBM Corp., Armonk, NY, USA). 

## Results

### Sample characteristics

The final sample included 683 adult cancer survivors (Table 1). Participants had a mean age of 65.0 ± 13.1 years, and 55.5% of the sample were females. The mean time since cancer diagnosis was 11.2 ± 10.7 years. Using a PHQ-9 cutoff score of ≥ 5, 28.1% (n = 192) of participants were classified as having clinically relevant depressive symptoms. Overall, 72.1% of participants had excess body weight (32.9% overweight and 39.2% obese), and 96.5% were citizens of the country. Indicators of socioeconomic position showed that 45.7% reported low annual income (< $34,000), while 28.6% and 25.7% reported middle- and high-income levels, respectively. Most participants had completed higher education (63.8%) and were married or living in a common-law relationship (60.9%). Lifetime alcohol use was reported by 72.6% of the sample. The distribution of cancer types is detailed in Supplementary Table 1.

**Table 1:** Characteristics of participants (n = 683)

Characteristic	n	(%)
Sex		
Male	304	(45.5)
Female	379	(55.5)
Citizenship		
American citizen	659	(96.5)
Non-American citizen	24	(3.5)
Marital status		
Married/common	415	(60.9)
Widowed	117	(16.9)
Divorced/separated	107	(15.6)
Never married	44	(6.6)
Education level		
Low education	108	(15.7)
High school	140	(20.5)
Higher education	435	(63.8)
Income level		
Low-income	313	(45.7)
Middle-income	196	(28.6)
High-income	174	(25.7)
BMI		
Normal	190	(27.8)
Overweight	225	(32.9)
Obese	268	(39.2)
Lifetime alcohol use		
Yes	496	(72.6)
No	187	(27.4)

Data are presented as absolute (n) and relative (%) frequencies

Regarding 24-h movement behaviors, more than half of the participants (78.5%, n = 536) met the MVPA threshold (150 min/week), while 21.5% (n = 147) accumulated less than 150 min/week. High sedentary time was also highly prevalent, with 56.4% (n = 385) spending more than 8 h/day in sedentary activities, whereas 43.6% (n = 298) spent less than 8 h/day. Regarding nightly sleep time, 53.0% (n = 362) of participants had non-recommended sleep duration, defined as fewer than < 7 or > 9 h/day per night, while 47.0% (n = 321) had a sleep time within the recommended range 7–9 h/day per night. In addition, 43.3% met the combined recommendation for being physically active and having low sedentary behavior (n = 296). A total of 37.3% met the combined criteria for being physically active and achieving adequate nightly sleep time (n = 255). In addition, 23.0% met the combined recommendations for low sedentary behavior and adequate nightly sleep time (n = 157). When considering all three behaviors simultaneously (physical activity, adequate sleep, and low sedentary time), 23.0% met the recommendations (n = 157).

### 24-h movement behaviors and depressive symptoms

**Fig. 1:**
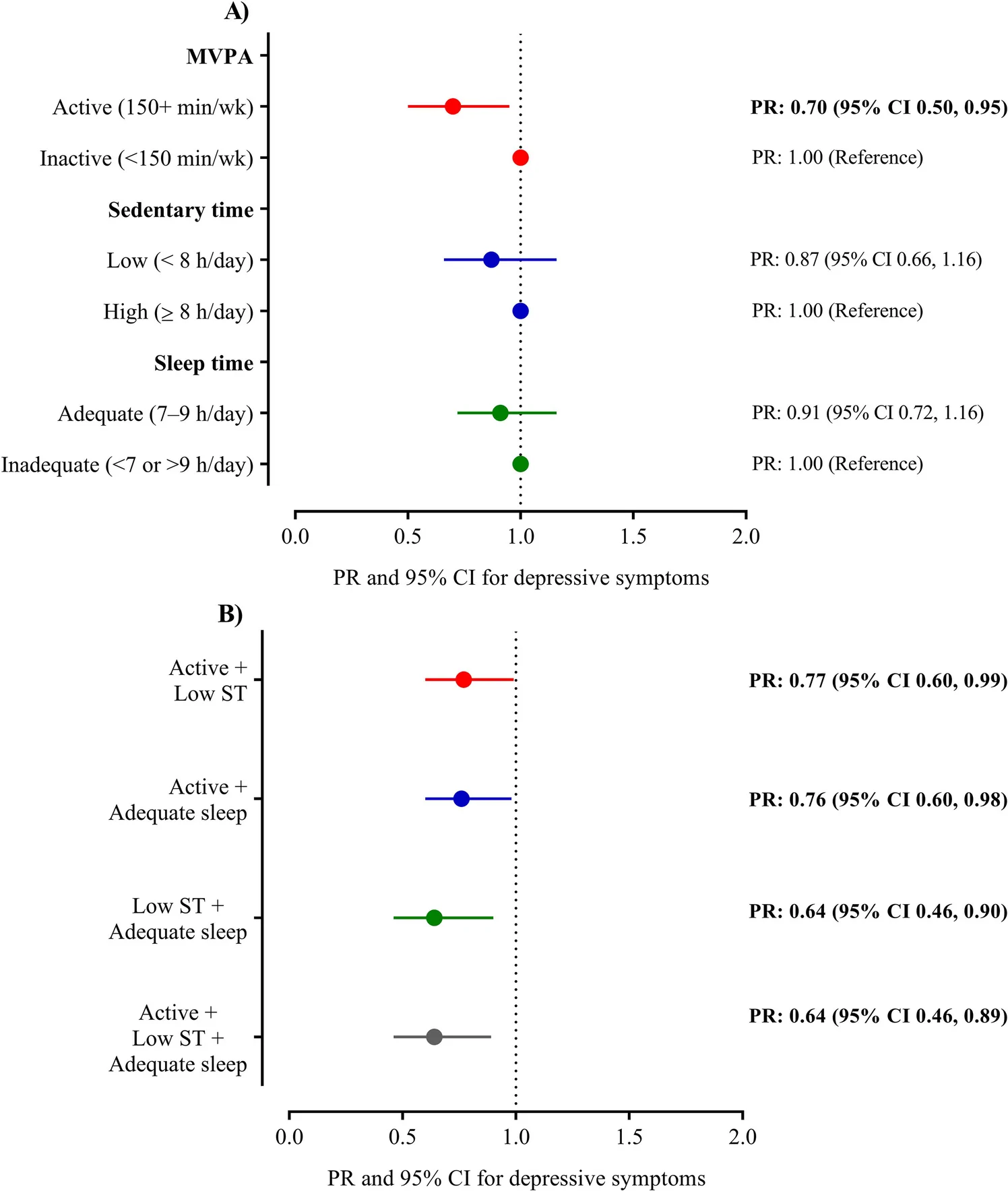
**A** Association between 24-h movement behaviors mutually adjusted and depressive symptoms in adult cancer survivors (n = 683). **B** Association between 24-h movement behaviors with different combinations and depressive symptoms in adult cancer survivors (n = 683). Data are presented as prevalence ratios (PR) with 95% confidence intervals (CI). Values in bold indicate statistically significant models (p < 0.05). This mutually adjusted and combined model included moderate-to-vigorous physical activity (MVPA), sedentary time (ST), nightly sleep time, age, sex, body mass index categories, income, educational level, and alcohol use

In the mutually adjusted model, MVPA was associated with lower depressive symptoms in cancer survivors (Fig. 1A). Cancer survivors considered active (+ 150 min/week) (PR = 0.70; p = 0.026), had a lower PR for having depressive symptoms. In contrast, sedentary time and nightly sleep time were not significantly associated to depressive symptoms (p > 0.05). For more details, see Supplementary Table 2. Findings were consistent across sensitivity analyses, with results for exclusion of non-melanoma skin cancer survivors presented in Supplementary Tables 4 and 5.

### 24-h movement behaviors combined and depressive symptoms

Cancer survivors who met recommendations for both MVPA and sedentary time showed a 23% lower prevalence of depressive symptoms compared with controls (PR = 0.77; p = 0.044). Those meeting recommendations for both MVPA and sleep time exhibited a 24% lower prevalence (PR = 0.76; p = 0.041). Additionally, those who met recommendations for sedentary time and sleep exhibited a 36% lower prevalence (PR = 0.64; p = 0.009). Moreover, meeting all three movement behavior recommendations was associated with a 36% lower prevalence of depressive symptoms (PR = 0.64; p = 0.009). For more details, see Fig. 1B and Supplementary Table 3. Findings were consistent across sensitivity analyses, with results for exclusion of non-melanoma skin cancer survivors presented in Supplementary Tables 4 and 5.

## Discussion

This study examined the association between accelerometer-measured 24-h movement behaviors and depressive symptoms in cancer survivors based on the Canadian 24-Hour Movement Guidelines. Since physical activity, sedentary time, and sleep time represent interdependent dimensions of human (non)movement across the 24-h cycle, this investigation may provide a greater understanding of the combined impact of these behaviors on depressive symptoms among cancer survivors. Our main findings were: i) higher MVPA were consistently associated with fewer depressive symptoms, whereas sedentary time and nightly sleep duration showed no such associations alone; ii) combinations of 24-h movement behaviors involving MVPA and sedentary time, MVPA and sleep time or sedentary and sleep time were associated with fewer depressive symptoms; iii) meeting all three (MVPA, low sedentary time, and adequate nightly sleep time), were consistently associated with lower depressive symptoms. Together, our findings indicate that meeting MVPA recommendations, individually and in combination with other 24-h movement behaviors, is associated with lower depressive symptoms in cancer survivors.

A substantial body of evidence indicates that MVPA has a strong beneficial impact on depressive symptoms in the general population [7]. However, this relationship remains underexplored among cancer survivors. To date, only a recent study involving females with breast cancer has examined this association using accelerometer-measured MVPA [5]. The authors reported an inverse relationship between MVPA and depressive symptoms, indicating that higher MVPA levels were associated with fewer depressive symptoms [5]. Our results support previous findings and add a novel contribution by providing new perspectives on the role of MVPA and combined sedentary time on depressive symptoms in cancer survivors. According to our findings, being physically active and having low sedentary time or adequate nightly sleep time are jointly associated with lower depressive symptoms in this population. This suggests that engaging in more than 22 min of physical activity per day, accumulating less than 8 h/day of sedentary time, or sleeping 7–9 h/day, rather than being inactive and/or highly sedentary or sleeping more than recommended, may be relevant to depressive symptoms among cancer survivors. These findings align with emerging evidence indicating that even low doses of daily physical activity reinforcing the public health message that any physical activity is better than none [33]. Also, when physical activity co-occurs with lower sedentary time and adequate nightly sleep time, synergistic mental health benefits have been observed, highlighting the importance of considering multiple movement behaviors rather than isolated domains.

Regarding sedentary time, our null findings partially contrast with previous studies in cancer survivors, suggesting that higher sedentary time may be inversely associated with happiness and, indirectly, with depressive symptoms [5, 34]. Other studies have reported that prolonged sedentary time is associated with higher depressive symptoms, potentially through bidirectional pathways [35]. However, evidence in cancer survivors remains limited, particularly when sedentary time is assessed objectively using accelerometry. Importantly, several studies examining accelerometer-measured sedentary time in cancer survivors have also reported null associations with depressive symptoms [5, 36], which is consistent with our findings. In contrast, some studies combining accelerometry with self-reported sedentary time have observed positive associations between sedentary time and depressive symptoms [6, 37]. One possible explanation for these inconsistencies is that accelerometer-measured sedentary time does not capture the context of this behavior. Certain types of sedentary activities (e.g., screen time, leisure activities, or work tasks) may be more strongly related to depressive symptoms than total sedentary time alone. Nevertheless, when combined with another positive behavior, such as being active or having adequate nightly sleep time, there is an association with lower depressive symptoms. Combining low sedentary time with adequate nightly sleep time may provide a greater reduction in symptom prevalence than combining low sedentary time with being active. These behaviors do not withdraw from each other. Still, the effect sizes in our study differed: the prevalence ratio was 23% for low sedentary time + being active, and 36% for low sedentary time + adequate nightly sleep time. Therefore, the lack of contextual detail in accelerometer data may partly explain the null findings observed in the present study. However, when sedentary time is combined with sleep, the pattern changes, suggesting that other behaviors are essential to potentiate one another. Future studies integrating device-based and self-reported measures of screen-based and other types of sedentary behaviors may help clarify these relationships.

Despite the well-established relationship between sleep time and mental health outcomes [38, 39]. Our findings do not indicate a significant association between nightly sleep time and depressive symptoms. The only association observed was when this sleep time behavior was combined with low sedentary time or being physically active. The mechanisms underlying sleep are multifaceted and may also be influenced by psychosocial factors such as family support, comorbidity, physical symptoms, treatment side effects, and stress related to the cancer experience [40–42]. Recommendations for sleep time in adults generally converge on the consensus that individuals should obtain between 7 and 9 h of sleep per night [43]. Deviations from this consensus may lead to several neurophysiological consequences, including disruptions in circadian rhythm and impairments in basic central nervous system functions [44], which can negatively affect overall health [44, 45]. Specifically, changes in sleep time may increase depressive symptoms [46, 47] and may even elevate mortality risk [48]. One possible explanation is that prolonged periods of inadequate sleep can give rise to sleep disorders characterized by difficulties initiating or maintaining sleep, which are strongly associated with greater severity of depressive symptoms [44].

The framework of the 24-h movement behavior cycle emphasizes achieving a balanced distribution of physical activity, sedentary time, and nightly sleep time. The Canadian guideline reports that adults and older adults should accumulate at least 150 min of MVPA per week, less than 8 h per day of sedentary activities, and sleeping 7–9 h/night [23]. Considering the combination of these behaviors, our study found that cancer survivors who engaged in more active patterns were less likely to experience depressive symptoms than those who were more inactive. In the combination of participants who met all three recommendations simultaneously, they were less likely to experience depressive symptoms compared with those who met only two movement behaviors. This pattern was observed in both the unadjusted and adjusted models. Acknowledging the cross-sectional nature of the study, it may be that adopting healthier movement behaviors after cancer treatment may help reduce psychological distress. Alternatively, it may be that those who experience heightened depressive symptoms are less likely to engage in MVPA.

The benefits of meeting the 24-h movement behavior recommendations are well established [23]. Within the 24-h movement behavior, MVPA, sedentary time, and sleep are interrelated, such that increases in one necessarily reduce time spent in the others. This integrative perspective suggests that mental health benefits may arise not only from engaging in MVPA but also from achieving a healthier balance across the full 24-h cycle. MVPA has well-known benefits for overall cardiovascular and metabolic health and can serve as a treatment and preventive strategy for several pathological conditions [49, 50]. Regarding neurobiological benefits, MVPA increased prefrontal cortex (PFC) oxygenation [51] and brain structure [52], which is associated with improved cognition, such as emotional control (emotional responses to adapt to situational demands), and ultimately improved mental health [53]. In addition, physical activity increases neurogenesis and enhances levels of important neurotrophic factors for mental health [54]. Physical activity also provides psychosocial benefits, such as improved quality of life and higher self-efficacy [55, 56], all of which may help alleviate depressive symptoms [57]. Similarly, reducing sedentary time can enhance emotional stability, which may help reduce depressive symptoms [58]. In addition, maintaining adequate sleep time may help prevent sleep disorders and promote a healthier hypothalamic–pituitary–adrenal (HPA) axis function, which, in turn, influences mechanisms related to emotional regulation [59]. Despite this, our mutually adjusted analyses identified MVPA as the key 24-h movement behavior component associated with depressive symptoms in cancer survivors. Whereas meeting all three behaviors, accumulating at least 150 min/week of MVPA, having low sedentary time (< 8 h/day), and obtaining adequate sleep (7–9 h/day), was associated with lower depressive symptoms in cancer survivors.

### Clinical implications

From a 24-h movement behavior perspective, MVPA, sedentary time, and sleep coexist within a finite daily cycle and influence one another. Achieving a healthier distribution of time across this cycle may support better mental health in cancer survivors. Combinations of behaviors involving MVPA, particularly when integrated with lower sedentary time, or lower sedentary time and adequate sleep time were associated with fewer depressive symptoms. Considering each movement alone, additional barriers to being physically active, including functional impairments and mood/stress-related and the prevalence of smoking, obesity, anxiety, depression, and lower self-efficacy, may limit one’s ability to be physically active [60]. This is an important issue, given that adherence to physical activity remains low, despite people’s awareness of its benefits for health [61]. Hence, we suggest that “any and all” physical activity should be encouraged. A simple message like ‘sit less and move more, more often’ [62] should be encouraged. To reduce sedentary behavior, simple strategies may be adopted throughout the day. For example, simply standing while working, using the phone, or while in group therapy might be helpful. Additionally, interrupting sedentary periods by standing/walking for 1–2 min every 30 min, may be positive for mental health [63]​. To improve sleep time, engaging in physical activity can be a valuable strategy, and incorporating mindfulness-based practices may also be highly effective in enhancing sleep quality [64]. In addition, emphasizing habits that regulate the circadian cycle and reduce nighttime interruptions, such as establishing consistent bedtimes and wake times [65], may help stabilize the sleep–wake rhythm. Therefore, adjusting the time spent in each behavior to remain within ideal levels (i.e., meeting the 24-h movement behaviors) is extremely important for reducing the likelihood of depressive symptoms.

### Limitations and strengths

This study has methodological limitations that should be mentioned. First, its cross-sectional design precludes causal inference. Second, the use of self-reported cancer diagnoses without detailed information on cancer type or characteristics (e.g., recurrence, prognosis, stage and treatment type) may limit the generalizability of the findings for this group. Additionally, limited information was available regarding treatment-related factors that may influence mental health outcomes. Third, we did not use any tool to assess different forms of sedentary activities, such as screen time, which limits our ability to further explore sedentary time. Nonetheless, these measures provide valuable perspectives on how various sedentary behaviors (e.g., screen time or work tasks) throughout the 24-h period may be associated with mental health. Finally, we acknowledge that the accelerometer-measured approach does not capture within-day or between-day compensatory behaviors following MVPA, nor the dynamic redistribution of time across behavior domains. The 24-h movement framework was therefore applied conceptually, as an organizing structure aligned with the Canadian guidelines. Conversely, this study presents several notable strengths, particularly in its real-world applicability, contributing to global efforts to evaluate 24-h movement behaviors in cancer survivors. The use of device instruments, considered robust and the gold standard for assessing movement behaviors, ensures a more precise quantification of the 24-h activity cycle. Moreover, these findings underscore the potential of this research to advance understanding of mental health symptoms in high-income countries where access to specialized mental health services may be limited.

## Conclusion

Our findings indicate that MVPA, rather than sedentary time or nightly sleep time, was the 24-h movement behavior associated with depressive symptoms among cancer survivors. Notably, associations were evident in those who met recommendations for MVPA and sedentary time or nightly sleep time, and sedentary time and sleep were associated with a lower prevalence of depressive symptoms. This association was strongest when all recommendations were met, suggesting that meeting all recommendations may be relevant to mental health in this population.

## Supplementary Information

Below is the link to the electronic supplementary material.

Supplementary file1 (DOCX 15 kb)

Supplementary file2 (DOCX 20 kb)

Supplementary file3 (DOCX 21 kb)

## Data Availability

The datasets used and analyzed during the current study are available from the corresponding author on reasonable request.
