# Supplementary material for: Associations of 24-h movement behaviors with depressive symptoms among cancer survivors: findings from the NHANES 2011–2014
Source: J Cancer Surviv. 2026 May 16;20(Suppl 2):2031–40. doi: 10.1007/s11764-026-02034-0 (PMC13744540; doi:10.1007/s11764-026-02034-0)
Supplement: Supplementary file 1 [file 11764_2026_2034_MOESM1_ESM.docx]

**Supplementary Table 1** - Detailed cancer type distribution (n = 683)

| **Cancer type** | **n** | **(%)** |
| --- | --- | --- |
| Bladder | 14 | (2.0) |
| Blood | 3 | (0.4) |
| Bone | 2 | (0.3) |
| Brain | 3 | (0.4) |
| Breast | 96 | (14.1) |
| Gynecological | 78 | (11.4) |
| Colon | 24 | (3.5) |
| Esophagus | 3 | (0.4) |
| Kidney | 10 | (1.4) |
| Leukemia | 3 | (0.4) |
| Lung | 18 | (2.6) |
| Lymphoma/Hodgkin | 12 | (1.8) |
| Melanoma | 37 | (5.4) |
| Pancreas | 2 | (0.3) |
| Prostate | 88 | (12.9) |
| Rectum | 5 | (0.7) |
| Skin non-melanoma | 92 | (13.5) |
| Soft tissue | 3 | (0.4) |
| Stomach | 5 | (0.7) |
| Testis | 5 | (0.7) |
| Thyroid | 13 | (1.9) |
| Multiple cancers | 167 | (75.5) |

Data are presented as absolute (n) and relative (%) frequencies.
