# Supplementary material for: Associations of 24-h movement behaviors with depressive symptoms among cancer survivors: findings from the NHANES 2011–2014
Source: J Cancer Surviv. 2026 May 16;20(Suppl 2):2031–40. doi: 10.1007/s11764-026-02034-0 (PMC13744540; doi:10.1007/s11764-026-02034-0)
Supplement: Supplementary file 2 [file 11764_2026_2034_MOESM2_ESM.docx]

**Supplementary Table 2**

Association between 24-hour movement behaviors and depressive symptoms in adult cancer survivors (n = 683)

|  | **Unadjusted model** | |  |  | **Adjusted model** | |  |
| --- | --- | --- | --- | --- | --- | --- | --- |
|  | **PR** | **(95% CI)** | **P-value** |  | **PR** | **(95% CI)** | **P-value** |
| **Moderate-to-vigorous physical activity** |  |  |  |  |  |  |  |
| Active (150+ min/wk) | 0.80 | (0.61, 1.04) | 0.104 |  | 0.70 | (0.50, 0.95) | **0.026** |
| Inactive (<150 min/wk) | 1.00 | Reference |  |  | 1.00 | Reference |  |
| **Sedentary time** |  |  |  |  |  |  |  |
| Low (< 8 h/day) | 0.88 | (0.69, 1.12) | 0.312 |  | 0.87 | (0.66, 1.16) | 0.371 |
| High (≥ 8 h/day) | 1.00 | Reference |  |  | 1.00 | Reference |  |
| **Sleep time** |  |  |  |  |  |  |  |
| Adequate (7–9 h/day) | 0.84 | (0.66, 1.08) | 0.183 |  | 0.91 | (0.72, 1.16) | 0.483 |
| Inadequate (<7 or >9 h/day) | 1.00 | Reference |  |  | 1.00 | Reference |  |

*Note*. Data are presented as prevalence ratios (PR) with 95% confidence intervals (CI). Values in bold indicate statistically significant models (p < 0.05). Adjusted models included moderate-to-vigorous physical activity, sedentary time, nightly sleep time, age, sex, body mass index categories, income, educational level, and alcohol use. All statistically significant adjusted models demonstrated adequate goodness of fit, as indicated by the Omnibus test (p < 0.05).

**Supplementary Table 3**

Association between 24-hour movement behaviors and depressive symptoms in adult cancer survivors (n = 683)

|  | **Unadjusted model** | |  |  | **Adjusted model** | |  |
| --- | --- | --- | --- | --- | --- | --- | --- |
|  | **PR** | **(95% CI)** | **P-value** |  | **PR** | **(95% CI)** | **P-value** |
| **Model 1** |  |  |  |  |  |  |  |
| Active + Sedentary time (< 8 h/day) | 0.87 | (0.68, 1.12) | 0.289 |  | 0.77 | (0.60, 0.99) | **0.044** |
| Control | 1.00 | Reference |  |  | 1.00 | Reference |  |
| **Model 2** |  |  |  |  |  |  |  |
| Active + Sleep time (7–9 h/day) | 0.76 | (0.58, 0.99) | **0.044** |  | 0.76 | (0.59, 0.98) | **0.041** |
| Control | 1.00 | Reference |  |  | 1.00 | Reference |  |
| **Model 3** |  |  |  |  |  |  |  |
| Sedentary (< 8 h/day) + Sleep time (7–9 h/day) | 0.69 | (0.50, 0.96) | **0.031** |  | 0.64 | (0.46, 0.90) | **0.009** |
| Control | 1.00 | Reference |  |  | 1.00 | Reference |  |
| **Model 4** |  |  |  |  |  |  |  |
| Meeting all 3 recommendations | 0.69 | (0.50, 0.96) | **0.031** |  | 0.64 | (0.46, 0.89) | **0.009** |
| Meeting ≤2 recommendations | 1.00 | Reference |  |  | 1.00 | Reference |  |

*Note*. Data are presented as prevalence ratios (PR) with 95% confidence intervals (CI). Values in bold indicate statistically significant models (p < 0.05). **Model 1:** Adjusted models included participants who met both the recommendations for moderate-to-vigorous physical activity and sedentary time, compared to controls who met one or none of these two movement behaviors. Covariates included age, sex, body mass index categories, income, educational level, and alcohol use. **Model 2:** Adjusted models included participants who met both recommendations for moderate-to-vigorous physical activity and for nightly sleep time, compared with controls who met one or neither of these movement behaviors. Covariates included age, sex, body mass index categories, income, educational level, and alcohol use. **Model 3:** Adjusted models included participants who met both recommendations for sedentary time and nightly sleep time, compared with controls who met one or neither of these movement behaviors. Covariates included age, sex, body mass index categories, income, educational level, and alcohol use. **Model 4:** Adjusted models included participants who met all three recommendations (moderate-to-vigorous physical activity, sedentary time, and nightly sleep time), compared to controls who did not meet all three movement behaviors. Covariates included age, sex, body mass index categories, income, educational level, and alcohol use. All statistically significant adjusted models demonstrated adequate goodness of fit, as indicated by the Omnibus test (p < 0.05).
